# Evidence base for non-genetic inheritance of environmental exposures in non-human animals and plants: a map of evidence syntheses with bibliometric analysis

**DOI:** 10.1186/s13750-022-00290-y

**Published:** 2023-01-06

**Authors:** Erin L. Macartney, Szymon M. Drobniak, Shinichi Nakagawa, Malgorzata Lagisz

**Affiliations:** 1 Evolution and Ecology Research Centre, School of Biological, Earth and Environmental Sciences, University of New South Wales, Sydney, NSW Australia; 2 Institute of Environmental Sciences, Jagiellonian University, Krakow, Poland

**Keywords:** Environmental effects, Scoping review, Inter-generational inheritance, Trans-generational inheritance, Maternal effects, Paternal effects, Systematic review

## Abstract

**Background:**

Direct effects of parental environment (particularly mothers) on offspring have been frequently demonstrated over the last decades. More recently ‘indirect’ non-genetic effects of ancestral environment and environmental effects through the patriline have been observed. Such research has captured the interest of many disciplines including biomedical science, toxicology, agriculture, and ecology and evolution due to the importance of understanding environmental effects on individual and population health. Consequently, the secondary literature, aimed at synthesizing non-genetic effects has also been increasing. The non-genetic inheritance secondary literature can be as diverse as the primary literature. Thus, there is a need to ‘map’ the non-genetic inheritance secondary literature to understand the state of the field and move forward in filling research gaps. Here, we ask four main questions: (1) What evidence exists on the impacts of non-genetic inheritance in non-human animals and plants across disciplines within the secondary ‘systematic-like’ (evidence synthesis) literature (2) What are the discipline-specific research patterns and gaps? (3) How connected is the literature (i.e., shared citations within and between disciplines, and collaborations between different countries)? (4) What is the overall quality of the non-genetic inheritance SR literature?

**Methods:**

We systematically searched for published and grey evidence syntheses on non-genetic inheritance in non-human animals and plants. We then extracted details pertaining to research topics and assigned each article to one of five disciplines (agriculture, biomedical science, ecology and evolution, toxicology, and cross-disciplinary research). We mapped within- and between- discipline research patterns through descriptive statistics and visualizations, and conducted a bibliometric analysis of the ‘connectedness’ of the literature (i.e., co-citation and collaboration networks). We also conducted a critical appraisal of the included articles.

**Results:**

We show that most evidence syntheses were in biomedical science and synthesized primary literature on rats and mice. Most evidence syntheses examined ‘direct’ effects of ancestral environment on descendants, particularly maternal dietary effects on offspring physiology and morphology. Ecology and evolution and cross-disciplinary evidence syntheses included the most diverse range of primary literature in their articles. We also show that most evidence syntheses have at least one author affiliated with an institution in the USA, and that the UK tends to form the most multinational collaborations. Toxicology evidence syntheses were least likely to cite studies outside of its own discipline. Lastly, we show where the quality of the non-genetic inheritance systematic-like literature could be improved.

**Conclusions:**

We have highlighted that certain areas of non-genetic inheritance are more frequently synthesised than others which may reflect a stronger interest in certain research topics at either the secondary or primary literature level. Presenting these research patterns and gaps in the literature that will not only make it easier to for researchers to understand the current state of the literature, but will also aid in bridging gaps between disciplines in the future. This will have substantial benefits for our understanding of non-genetic inheritance, with implications for many research fields, including climate change research, ecological and evolutionary theory, and understanding the effects of environmental pollutants on population health. It will also help policy makers identify relevant literature to inform policies, especially related to the negative impacts of environmental factors across generations.

**Supplementary Information:**

The online version contains supplementary material available at 10.1186/s13750-022-00290-y.

## Background

**Fig. 1:**
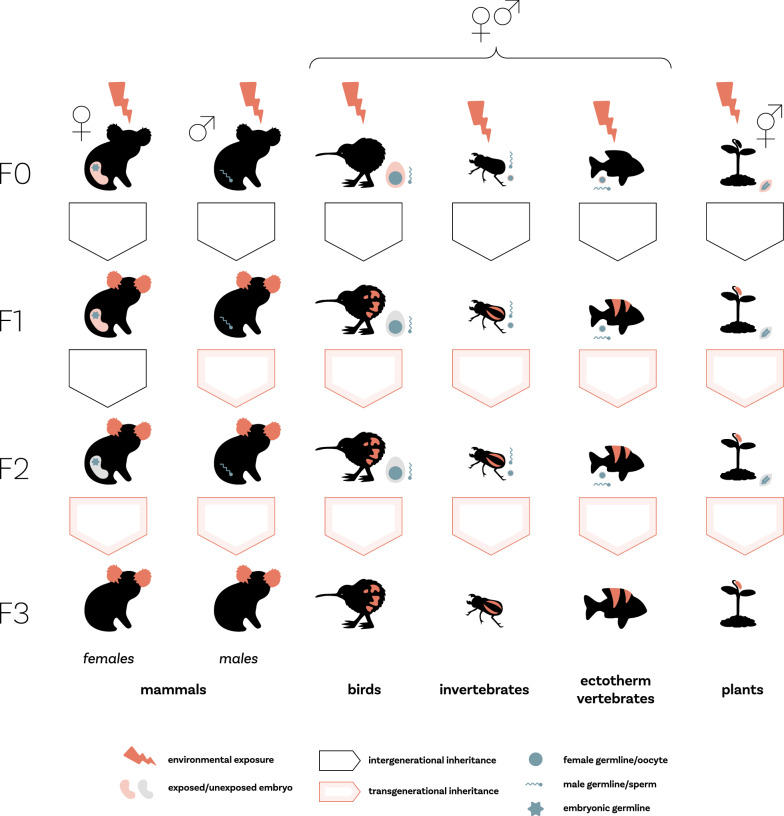
Figure adapted from our published protocol [19]. Inter-generational effects occur in the F1 and F2 descendants through the matriline of mammals as the F2 germline within the F1 embryo is exposed to environmental influences during F0 pregnancy. Inter-generational effects also occur in the F1 if the impacts of the environmental exposure (e.g., through epigenetic modifications) are passed through the patriline of any taxonomic group or the matriline of taxonomic groups other than mammals (where inter-generational effects also occur in the F2). Trans-generational effects occur in the F3 and further if non-genetic effects are conferred through the matriline in mammals and the F2 and further if the non-genetic effect is conferred through the patriline or through the matriline of non-mammals. Note that Fig. 1 is only used to provide a general overview and does not show all the taxonomic groups in which non-genetic inheritance can occur

The parental environment can be a potent force influencing offspring phenotype beyond that of their genetic contributions [1, 2]. Such environment-induced non-genetic inheritance is widely observed through direct maternal provisioning to the developing embryos [3–5]. For example, mothers that are malnourished prior to or during pregnancy can directly alter the health and fitness of their offspring [6, 7]. A frequently referenced example in humans is the effects of the “Dutch Hunger Winter” of 1944–1945 where mothers that were subjected to famine had children with altered physiology and metabolism that made them more prone to type-2 diabetes and cardiovascular disease [8, 9]. Such effects of maternal diet have also been widely shown in animals, particularly in rodents (e.g., [5, 8–10]). Similarly, it is now accepted that the paternal environment can also directly alter offspring quality [11–13]. This can either occur through direct provisioning of nutrient-rich ejaculates in some species [14, 15] or through epigenetic changes within the sperm cells/seminal fluid [16–18]. Such direct effects are commonly referred to as ‘inter-generational effects’ [note that female mammals can also directly confer non-genetic effects to their grand offspring through the maternal line (see Fig. 1)].

More recently, non-genetic inheritance has been shown to occur beyond the generation directly exposed to parental conditions as an embryo or germ cell, commonly referred to as ‘trans-generational effects’ (see Fig. 1) (e.g., [14–18]). For example, in humans, the children of fathers whose mothers were subject to famine during the Dutch Hunger Winter had higher body weights and body mass indices compared to the children whose grandmothers (through the patriline) were not exposed to famine [20]. Again, such trans-generational effects have also been demonstrated in animals. For example, grandoffspring whose grandparents consumed obesogenic diets can be more prone to metabolic syndromes in rodents [21], and warmer water temperature can result in altered physiology in multiple generations in the tropical damselfish, *A. polycanthus* [22]. Such trans-generational effects indicate that at least some environment-induced information remains stable across generations. Both inter-generational and trans-generational effects are broadly referred to as ‘non-genetic inheritance’ and understanding such effects is highly important for a broad spectrum of research areas, including our understanding of inherited diseases and syndromes [23–25], the effects of climate change and environmental pollutants on individual and population health [22, 26, 27], as well as making further developments in ecological and evolutionary theory [2, 28, 29].

In recent decades there has been a rapid accumulation of primary studies examining non-genetic inheritance within multiple research disciplines. Consequently, there has also been an increase in secondary studies (i.e., literature syntheses). Primary studies are incredibly important for showing specific non-genetic effects (e.g., through experiments) and building knowledge foundations. Once enough primary literature has amassed, researchers can then transition into synthesizing this literature to determine the universality of such effects. In particular, the use of systematic-like reviews (see below for our definition; hereafter “evidence syntheses”), including quantitative analyses such as meta-analyses, are considered a highly effective and robust method for determining the generalizability of certain effects and highlighting potential drivers of variation [30, 31]. However, similarly to the primary literature, the questions addressed through evidence syntheses often vary in scope and can be discipline-specific. For example, toxicological and biomedical studies frequently ask highly specific questions such as the effects of certain environmental stressors or pollutants on specific offspring traits. These studies are often conducted on a taxonomically narrow group of study species such as rodents (a common preclinical study system, especially in biomedical science). In contrast, ecology and evolutionary studies frequently synthesize studies across a broad range of environments, offspring traits, and species [32].

Moreover, evidence syntheses may be disparate in numerous other ways, such as the life stage of parents/ancestors exposed to the environment of interest and the sex of the offspring/descendants. While these differences in scope are important for addressing discipline-specific questions, they can make it difficult to determine overarching patterns across disciplines. Beyond differences in scope, evidence syntheses can vary in the terminology used. For example, terms such as ‘inter-generational inheritance’ and ‘trans-generational inheritance’ are sometimes used interchangeably whereas at other times these terms are used with strict definitions (as in Fig. 1) [1]. Such differences in terminology can also make identifying relevant evidence syntheses difficult, thus, there is a need to make identifying relevant literature easier.

## Objectives

Here, we map and conduct a bibliometric analysis of the non-genetic inheritance evidence synthesis literature by identifying evidence syntheses that included primary studies on the effects of F0 (ancestral) environmental exposures on descendants (≥ F1) in non-human animals and plants (see Table 1). Our primary objective (question one) followed by three secondary objectives (questions two to four) are: (1) What evidence from evidence syntheses exists on the impacts of non-genetic inheritance in non-human animals and plants across disciplines, such as the most common types of environmental exposures and descendant traits examined, and are there any gaps in the literature (see Table 1 and the methods section for our PECO elements)? (2) Are there discipline-specific research patterns and terminology use, including commonalities and disparities, between disciplines? (3) How are authors of the included articles connected across different countries and how is the literature connected between disciplines? (4) What is the reliability of the included evidence syntheses?

**Table 1:** Scope of our map of evidence syntheses according to PECO framework

	Included	Excluded
Populations	• Evidence syntheses that included experimental studies on non-human species with clear separation of generations	• Evidence syntheses solely on humans or species that do not have clear separation of generations (e.g., reproduce through budding, fragmentation, or vegetative propagation) • Evidence syntheses that only included correlational studies (i.e., did not include experimental studies)
Exposures	• Evidence syntheses that included F0 environmental exposures that fitted within the following categories: (1) Diet (2) Human-induced pollutants/toxins (3) Natural variation in environmental composition (e.g., minerals and elements) (4) Psychological stress (5) Temperature (6) ‘Human health risk’ (e.g., tobacco and alcohol) (7) Population demographics (e.g., population density and sex ratio) (8) Light and/or photoperiod (9) Other	• Evidence syntheses that focused exclusively on human-related therapeutics/ pharmaceutical drugs such medicines and physical procedures (note that pharmaceuticals were included if they were within the context of environmental pollutants) • Evidence syntheses that focused exclusively on F0 polyandry or genetic inheritance
Comparators	• Evidence syntheses that included a control environment (e.g., standard laboratory conditions) or two ‘levels’ of the same environment (e.g., warm versus cold temperature)	• Evidence syntheses that exclusively focused on different populations so effects could not be clearly attributed to specific environmental differences • Evidence syntheses that only included correlational primary studies (i.e., did not include experimental studies)
Outcomes	• Evidence syntheses that included post-embryonic traits in the F1 or subsequent generations • Evidence syntheses that included outcomes fitted into the following categories: (1) Physiological (2) Morphological (3) Reproductive (4) Life-history (5) Behavioral (6) Molecular (7) Other	• Evidence syntheses that only focus on F0 fertility and fecundity such as offspring number

Note that a few evidence syntheses included some primary studies that did not meet our eligibility criteria (e.g., included studies on humans) but these articles were included in our map if the article did not exclusively focus on primary studies that did not meet our criteria (i.e., if it included mostly primary literature that met our selection criteria)

Addressing these questions is integral for advancing our understanding of within and between discipline research patterns, including the types of questions and topics examined, the terminology used, as well as quality (i.e., reliability) of research. It is also important for highlighting gaps in the literature, disciplines and countries that are conducting the most research, and areas where reliability and transparency can be improved. Overall, this work will aid researchers across disciplines and policymakers in planning future research directions, improve cross-disciplinary communication, and make policy decisions relating to the effects of environmental exposures across generations.

## Methods

We have followed the *RepOrting standards for Systematic Evidence Syntheses* (ROSES) for systematic map reports (adapted for mapping secondary literature) (Additional file 1). Please see [19] for our published protocol.

### Deviations from the protocol

We have adhered to our published protocol as closely as possible [19] but have made a few adjustments that allowed us to address our objectives and visualize our results more clearly. In particular, we have visualized discipline-specific research patterns using heatmaps rather than stacked bar charts, we have presented a phylogenetic tree of the species from the primary literature included in the evidence syntheses (including bar charts to show the frequency of each species across evidence syntheses), and grouped all included species into taxonomic groups (i.e., plants, reptiles, birds, mammals, etc.) rather than vertebrates versus invertebrates. Additionally, we reported some results in in the Open Science Framework found at https://osf.io/8q3a9/ rather than the main text if it did not seem integral to addressing our objectives.

### Searching for articles

We searched Topics (title, abstract, keywords) of three broad-coverage databases—Scopus, ISI Web of Science Core Collection, and PubMed (accessed through the University of New South Wales, Sydney) for systematic-like reviews (i.e., evidence syntheses; see below for definition of evidence synthesis) published up until the 23rd of November 2021 (i.e., the date the searches were conducted). We used a search string made of three groups of keywords: (1) those related to non-genetic inheritance (e.g., maternal, paternal, non-genetic inheritance, inter-generational, trans-generational), (2) those related to SR type (e.g., systematic review with or without meta-analysis), and (3) exclusion keywords filtering out most human-centered articles (e.g., men, women, person, worker, patient) as a majority of these studies are purely correlational and we focused on evidence syntheses that synthesized direct manipulations of the environment (most biomedical studies are experimental studies on rodents) (see Additional file 2 for exact search strings for each database). This search string was tested against a set of relevant benchmark articles (found in our protocol [19]) to ensure that our search was comprehensive.

We also searched titles in the academic grey literature (limited to Ph.D., Masters and Honours theses) using the Bielefeld Academic Search Engine and keywords relating to non-genetic inheritance ‘(maternal OR paternal OR non-genetic OR nongenetic OR inter-gen OR intergen OR trans-gen OR transgen) and review type (systematic OR meta-analysis OR metaanalysis)’. Then, we conducted backwards and forwards searching (‘snowballing’) of the cited and citing literature using the set of relevant studies detected from our database and grey literature searches. Thus, our search is anticipated to be comprehensive, although we did not include field-specific databases.

All searches were conducted in English but we included additional languages during our screening stage (see ‘Article screening’ below). Please see our published Protocol for an estimate of the comprehensiveness of the search [19].

### Article screening and study eligibility criteria

We used Rayyan QCRI [31] for abstract and full-text screening following the decision tree (Additional file 2: Fig. S1) and our PECO (Populations, Exposures, Comparators, Outcomes) framework for eligibility (Table 1). In addition to falling within our PECO framework, all studies must be within the ‘family’ of systematic reviews (i.e., ‘systematic-like’, hereafter referred to as “evidence syntheses”) [33], meaning that all studies systematically (as opposed to ad hoc) searched the literature for relevant primary studies and screened the studies using selection criteria. Evidence syntheses may be reported in any format, including narratives, formal and informal meta-analysis, maps, rapid and scoping reviews, or equivalent.

ELM and ML independently screened all abstracts and full-texts, and any conflicts were resolved by discussion and referring to the decision tree (see Additional file 2: Fig. S1). Conflicts did not exceed 10% of the articles screened.

We limited the language of the included studies to English, Polish, Russian, and Japanese during screening as these are the languages that we understand. However, we only detected studies in English and acknowledge that this may create a language bias in the evidence syntheses included in our work.

We note that we did not include evidence syntheses that focused solely on humans as human studies are predominantly correlational (the ancestral environment is not directly manipulated while controlling for other variables). We are likely to have captured many evidence syntheses that examine environmental effects that are also relevant to humans via rodent-based pre-clinical study systems, especially in biomedical science. However, we also note that we did not include evidence syntheses that focused purely on human therapeutics (i.e., medications), even if performed on animals, unless they were examined in the context of environmental pollution and toxicology. This is because pre-clinical studies examining the effects of therapeutics usually have the intention of only being applicable to humans and thus, are not widely applicable to any other species and are therefore out of the scope of this work.

### Data coding

For each SR that met our article screening criteria, we manually extracted details regarding: (1) the species and broad taxonomic group(s) of interest that the SR included (note that we did not use the formal taxonomic ranks such as Phylum or Class, but instead divided taxa into easily recognizable biological groups such as ‘plants’, ‘mammals, ‘birds’, etc.), if the SR included inter- or trans-generational effects, and if the terminology used to describe the non-genetic effects matched our definition in Fig. 1 (i.e., matched our definition of inter- and trans-generational effects), (2) details regarding the F0 exposure such as the type of exposure, the life stage of ancestral exposure such as during gestation etc., and the F0 sex/transmission mode (e.g., matriline or patriline), and (3) descendent outcomes such as the traits influenced by the ancestral exposure, the sex of the descendants, and if the ancestral environmental exposure was predicted to have negative or positive/beneficial effects on offspring. Each SR was also manually assigned into one of the five broad disciplines (based on the topic and journal of publication): agriculture, biomedical science, cross-disciplinary research, ecology and evolution, and toxicology, to allow for assessment of discipline-specific patterns.

We then downloaded bibliometric information, including country affiliations, and cited and citing literature, of the included articles directly from Scopus.

ELM manually extracted all data and ML cross-checked 20% of included articles. Any discrepancies or areas that were difficult to categorise (e.g., if an article did not easily fit within a discipline category) were discussed and resolved (discrepancies did not exceed 5%). See Additional file 4 for all extracted data and meta-data, https://osf.io/8q3a9/ also contains a relational database.

### Study validity assessment

We conducted a critical appraisal of the quality (i.e., rigor and transparency of methods, and risk of bias) of the included evidence syntheses following the *Collaboration for Environmental Evidence Synthesis Assessment Tool* (CEESAT) [34] Version 2.1. ELM assessed all included evidence syntheses (ELM was not an author on any included articles) and ML crosschecked 20% of included evidence syntheses (not including the articles that ML authored).

### Data mapping

All mapping (descriptive statistics and figures) was completed in the R Statistical Environment using RStudio version 2021.09.0 [35]. See Additional file 4 for data and https://osf.io/8q3a9/ for all code. The *ape* [36] and *rotl* [37] packages were used to create a phylogenetic tree of all the included species, the package *bibliometrix* [38] was used for much of the bibliometric analysis, and the packages *ggplot2* [39] and *circlize* [40] for visualizations.

## Review findings

### Review descriptive statistics

**Fig. 2:**
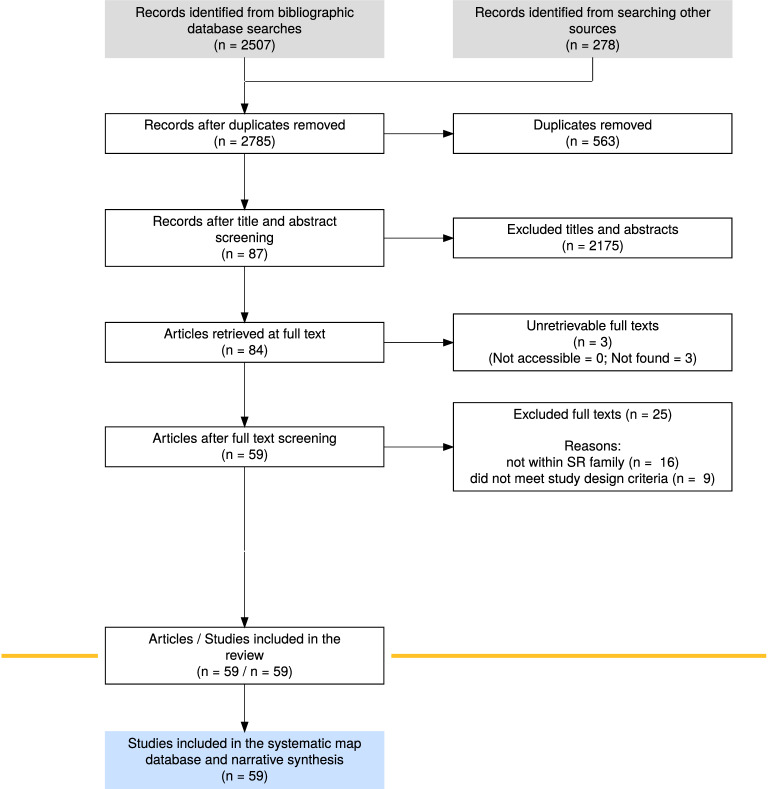
ROSES flow chart showing the number of articles included and excluded during each stage of the screening process

Searching Scopus, ISI Web of Science, PubMed and Bielefeld Academic Search Engine resulted in 2035 unique bibliographic records. After screening titles, abstracts, and keywords, we excluded 1955 articles for not meeting our abstract screening criteria (see Additional file 2: Fig. S1 for screening criteria) and accepted 80 articles for full-text screening (Fig. 2). During full-text screening, we excluded 25 articles for not meeting our full-text screening criteria (see Additional file 2: Fig. S1) and accepted 55 articles for inclusion in our map of evidence syntheses (Fig. 2). We then conducted backwards and forwards searches of the cited and citing literature of the 55 accepted full-text evidence syntheses. This resulted in a further 227 articles for abstract screening after duplicate removal. We then excluded 220 of these during title, abstract, and keyword screening and accepted 7 articles for full-text screening. During full-text screening, we excluded 3 articles and accepted an additional 4 articles (Fig. 2). Overall, we included 59 evidence syntheses in our map of evidence syntheses and bibliometric analysis (see Additional file 4 for a list of included articles and Additional file 2 for a list of excluded articles and reasons for exclusion).

### Mapping the quantity of studies relevant to the questions

#### Question one: evidence across disciplines

**Fig. 3:**
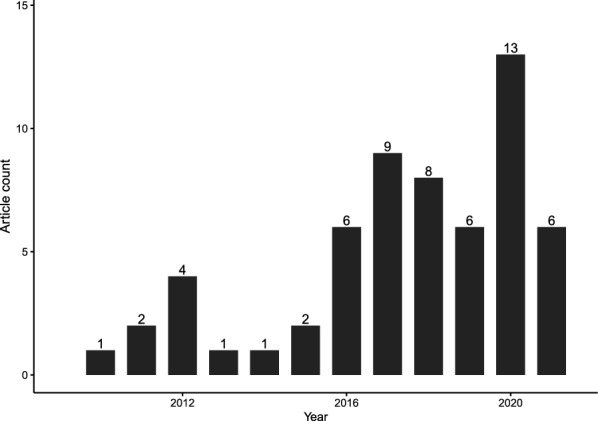
Bar chart showing the number of evidence syntheses published per year. Note that evidence syntheses from 2021 were only included up until the 23rd of November (when we conducted our literature search)

We show that evidence syntheses of the non-genetic inheritance literature are a relatively new research approach, with the first relevant evidence synthesis published in 2010 [41] (Fig. 3). Since then, there has been a steady increase in the number of evidence syntheses published per year (with some down-turns) and the highest number of evidence syntheses published in 2020 (13 articles) (Fig. 3). While some forms of non-genetic inheritance have been recognized for many decades [42], other areas of relevant primary research are relatively new. For example, studies examining non-genetic effects conferred through the patriline (“paternal effects”) and trans-generational effects beyond the F1 (beyond the F2 in female mammals; see Fig. 1) generation [43–48]. This increase in primary research has been spurred on by the discovery of non-genetic molecular mechanisms, such as epigenetic factors, which can confer non-genetic effects beyond ‘direct’ effects of parental condition on gametes and developing embryos [47, 49–53]. Also, given recent advances in systematic techniques, particularly in meta-analysis and for disciplines beyond biomedical science [33, 54–57], as well as a general increase in the number of articles published per year across fields [58] it is apt that the evidence syntheses of the non-genetic inheritance literature have only started to accumulate just over the last decade.

**Fig. 4:**
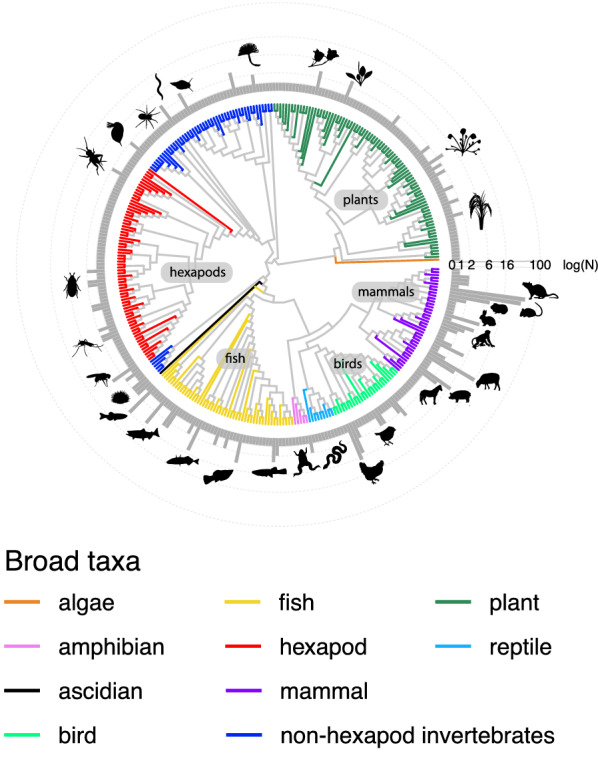
Phylogenetic tree of the species from the primary literature included in the evidence syntheses. Bars represent how many evidence syntheses (log10 transformed) included a given species (silhouettes of species only included for some of the most common species). Colours represent broad taxonomic groups (see legend). See Additional file 4 for a full list of species

The included evidence syntheses in our map covered primary literature on a wide range of species (335 species), across 18 broad taxonomic groups (Fig. 4). Interestingly, plants had the widest species diversity in the included articles (Fig. 4). However, all the included plant species came from two evidence syntheses [59, 60]. There were also some taxonomic groups that had a narrow diversity of species and a low frequency of evidence syntheses including species within these groups (e.g., many marine invertebrates, reptiles, and amphibians) (Fig. 4). While mammals were not the most diverse species group included in the articles, certain mammal species were by far the most common study species included in the Evidence syntheses. In particular, primary studies on rats (*R. norvegicus*) and mice (*M. musculus*) were the most frequently included in the evidence syntheses (86% and 71% respectively) (Fig. 4).

**Fig. 5:**
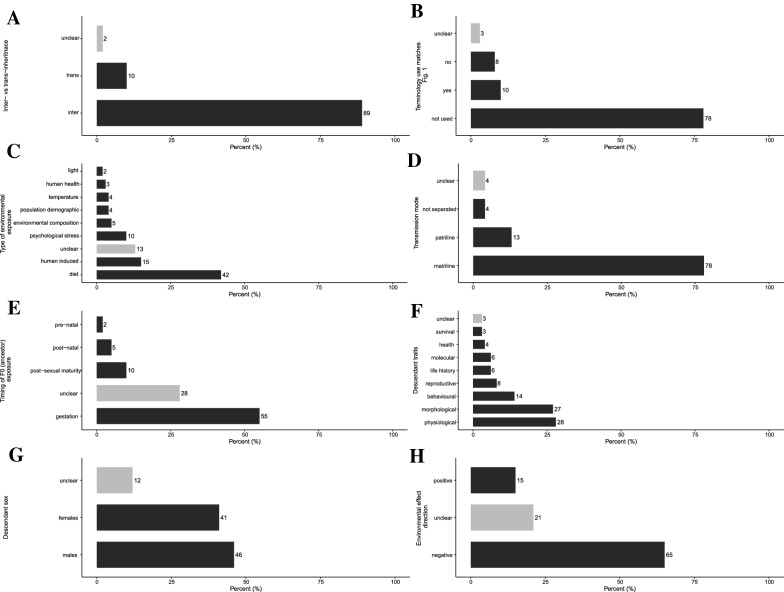
Bar charts showing **A** the proportion of evidence syntheses that examined inter- versus trans-generational inheritance (please also refer to Fig. 1), **B** if the terminology referring to non-genetic inheritance matched our definition provided in Fig. 1, **C** the type of environmental exposure, **D** the mode of transmission (i.e., through the matriline or patriline), **E** the timing of F0 (ancestral) environmental exposure, **F** the type of descendant traits, **G** the sex of descendants, and **H** the expected ‘direction’ of environmental effects on offspring (i.e., if the environment is expected to have a positive/beneficial effect or negative effect on offspring). Light grey bars represent the number of articles that did not clearly fit into any of the other categories (i.e., “unclear”)

There were also some clear biases in the research focus of the literature included in the evidence syntheses. 89% of articles examined inter-generational effects (i.e., ‘direct’ effects of ancestral environment on the gametes and developing embryos of descendants; see Fig. 1) compared to trans-generational effects (i.e., stable transfer of environmental induced phenotypes beyond the generation ‘directly’ exposed to ancestral environment; see Fig. 1) (Fig. 5A). However, a large majority (79%) of articles did not use the terms, “inter-generational” or “trans-generational” in their articles (Fig. 5B). Most (49%) evidence syntheses focused on primary studies that manipulated ancestral (F0) diet (Fig. 5C) and 78% of the environmental manipulations occurred in the matriline (Fig. 5D), and 55% of environmental exposures occurred during gestation (Fig. 5E). The two most common descendant trait categories were physiological traits (e.g., immune function and hormone concentrations) (28%) and morphological traits (e.g., birth weight and anogenital distance) (27%) (Fig. 5F) with approximately equal numbers of studies synthesized for male and female offspring (46% and 41% respectively) (Fig. 5G). 65% of environmental effects were predicted to have negative consequences on the offspring (Fig. 5H).

#### Question two: discipline-specific research patterns

**Fig. 6:**
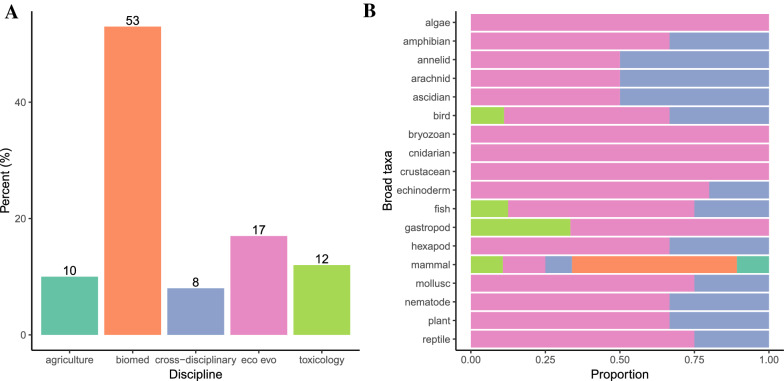
Bar chart showing **A** the percent of evidence syntheses from each discipline, and **B** the proportion of evidence syntheses within each discipline that included primary studies on each of the broad taxonomic groups. Colors represent disciplines where dark green = agriculture, orange = biomedical science (biomed), purple = cross-disciplinary, pink = ecology and evolution (evo evo), lime green = toxicology

Biomedical science made up just over half of the included evidence syntheses (53%), followed by ecology and evolution (17%), toxicology (12%), agriculture (10%), and then cross-disciplinary articles (8%) (Fig. 6A). Ecology and evolution articles included the widest diversity of broad taxonomic groups (spanning all of them), followed by cross-disciplinary studies (Fig. 6B). Agriculture and biomedical science had the narrowest diversity of taxonomic groups in their evidence syntheses as they only included primary studies on mammals (Fig. 6B). Given that biomedical science made up over 50% of the included evidence syntheses (Fig. 6A), and the pre-clinical nature of many biomedical studies (i.e., they often address narrow/highly specific questions that can be extended to humans), it makes sense that rats and mice were also the most common species included in the evidence syntheses (Fig. 4) as these species are the most frequently used as a pre-clinical study system [61]. In contrast, ecology and evolution and cross-disciplinary evidence syntheses often ask broad-scope questions that are generally applicable across species, and this is reflected in the broad range of taxonomic groups included in the evidence syntheses of these disciplines. Mammals were the only taxonomic group that all disciplines included in their evidence syntheses (Fig. 6B).

**Fig. 7:**
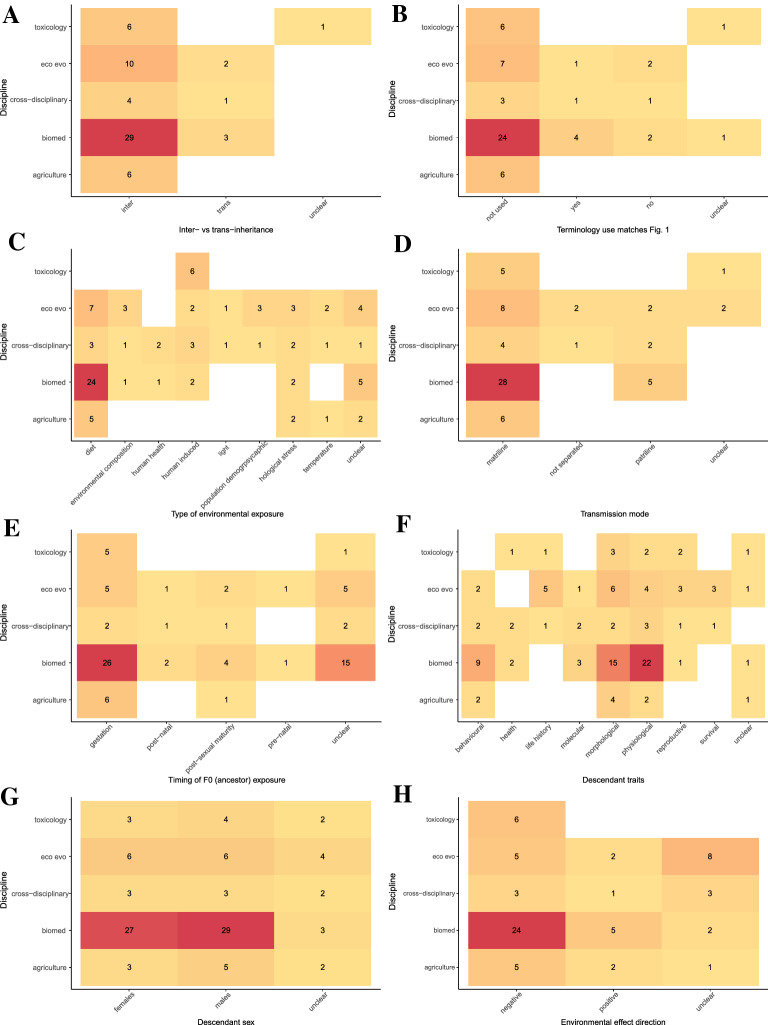
Heat maps showing the number of evidence syntheses within each of the five disciplines that examined **A** inter-versus trans-generational inheritance (also refer to Fig. 1), **B** if the terminology referring to non-genetic inheritance matched our definition provided in Fig. 1, **C** the type of environmental exposure, **D** the mode of transmission (i.e., through the matriline or patriline), **E** the timing of F0 (ancestral) environmental exposure, **F** the type of descendant traits, **G** the sex of descendants, and **H** the expected ‘direction’ of environmental effects on offspring (i.e., if the environment is expected to have a positive/beneficial effect or negative effect on offspring). Colors go from light (lowest number of articles) to dark (highest number of articles)

Evidence syntheses from all disciplines consistently examined inter-generational effects more often than trans-generational effects (Fig. 7A; see also https://osf.io/8q3a9/ Additional file 5: Fig. S4 for filial generations). However, the evidence syntheses seldom used the terms “inter-generational” or “trans-generational” within their articles. (Fig. 7B). Toxicology articles frequently examined human-induced environmental factors such as pollutants whereas the other four disciplines more frequently focused on dietary effects (Fig. 7C). Furthermore, cross-disciplinary articles included the widest range of environmental exposures, followed by ecology and evolution which included all environmental exposure categories apart from human health-related factors such as alcohol and drugs (Fig. 7C). All disciplines examined non-genetic effects through the matriline and exposure during gestation more frequently than through the patriline and other life stages (Fig. 7D, E), with biomedical science and ecology and evolution examining the widest diversity of exposure timings (Fig. 7E). Biomedical science and cross-disciplinary articles included physiological offspring traits more commonly in their evidence syntheses compared to agriculture, ecology and evolution, and toxicology that more frequently included morphological traits (Fig. 7F). Cross-disciplinary articles, followed by ecology and evolution included the widest diversity of descendant traits in their evidence syntheses; ecology and evolution articles did not include any traits related to descendant health (Fig. 7F). All disciplines (approximately) equally included responses on male and female descendants (Fig. 7G). Most disciplines expected negative effects of environmental factors on offspring, apart from ecology and evolution evidence syntheses where it was more common that effect directions were unclear (Fig. 7H). Again, these patterns likely reflect the scope of the questions asked by different disciplines. For example, the obesity epidemic induced by ‘Western’ or ‘cafeteria’ diets is likely driving the large number of biomedical science evidence syntheses on the effects of parental diet (particularly during gestation) on offspring physiology [4, 21, 62, 63]. Furthermore, agriculture is often concerned about gestational effects on offspring to ensure the health and fitness of livestock [64–68] and stability/predictability of artificially selected productivity-related traits. In contrast, ecology and evolution and inter-disciplinary studies are often interested in the generality of non-genetic effects across environmental exposures and traits to inform evolutionary and ecological theory [60, 69]. Similarly, the unclarity in expected direction of effects in ecology and evolution evidence syntheses is likely due to the broader hypotheses regarding environmental effects (e.g., ‘anticipatory effects’ [60, 69]) where predictions regarding if a particular environment is beneficial or likely to have negative consequences are less clear.

#### Question three: bibliometric analysis

**Fig. 8:**
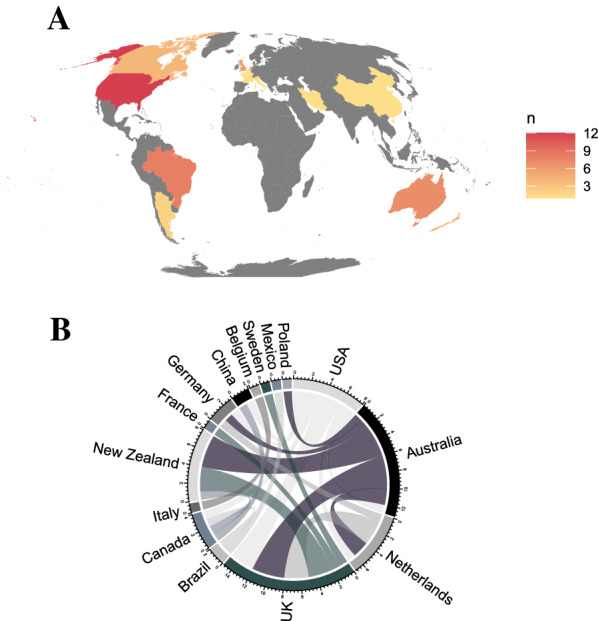
Geography of evidence syntheses authorships and collaborations. **A** Heat map of the world showing the number of evidence syntheses affiliated with each country (grey = no evidence syntheses affiliated with a country), **B** and a chord diagram of the collaborations between affiliate countries

The United States of America (USA) is the most prolific producer of the included evidence syntheses (i.e., authors of the included articles were affiliated with the USA), followed by Brazil, the United Kingdom (UK), Australia, New Zealand, and Canada (Fig. 8A). However, the UK tended to collaborate the most with other countries, followed by Australia, New Zealand, and the USA (Fig. 8B). While Brazil has been productive in publishing evidence syntheses in non-genetic inheritance, this country has not been involved in many inter-country collaborations (Fig. 8A, B). Most affiliations of the authors of the included evidence syntheses are from developed countries, with a trend towards English speaking countries. For example, we did not find any evidence syntheses authored by researchers from Africa, South-East Asia, Central America, or Eastern Europe (Fig. 8A). Such a bias is likely due to greater funding opportunities in developed countries and a greater propensity for evidence syntheses to be published in English [70, 71]. However, we acknowledge that a trend towards a bias in evidence syntheses from English speaking countries may also be due to a language bias in our search terms [72]. This bias towards research from developed and mostly English-speaking countries has also been noted in other research fields [73–75] and there is now a movement to try to shift research to be more globally diverse [76] which can provide more inclusive perspectives beyond that of colonial and primarily English-speaking counties [72, 77]. One benefit of evidence syntheses is the relatively lower research costs compared to primary research that often involves expensive laboratory and field work. Therefore, secondary research, including evidence syntheses in non-genetic inheritance, is ripe for filling the gaps in the literature pointed out above by scientists that may have limited funding (albeit funding is still required to cover journal subscriptions and potentially publication fees).

**Fig. 9:**
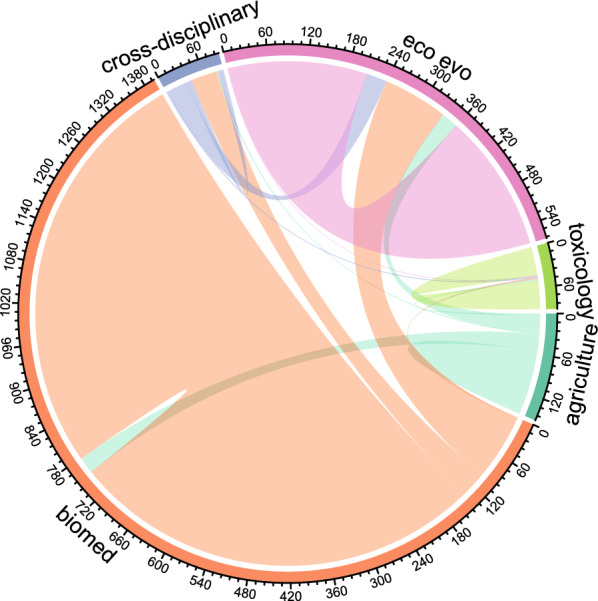
Disciplinary connectedness of evidence syntheses. Chord diagram of the shared citations of the included evidence syntheses within and between disciplines where the numbers indicate the number of shared citations and dark green = agriculture, orange = biomedical science (biomed), purple = cross-disciplinary, pink = ecology and evolution (eco evo), lime green = toxicology

When examining the shared citations (‘co-citations’, i.e., the references shared between two included evidence syntheses), we show that 84% of the shared citations from cross-disciplinary evidence syntheses were also cited by evidence syntheses from other disciplines (Fig. 9). Ecology and evolution and agriculture articles shared between 43% and 53% of their co-citations with other disciplines (Fig. 9). Toxicology and biomedical science largely tended to cite literature within their own field with both disciplines sharing less than 20% of their co-citations with other disciplines (Fig. 9) (16% and 18% respectively). Toxicology articles shared the lowest proportion of their shared citations with other disciplines (Fig. 9). This is surprising given that toxicology-related evidence syntheses were generally interested in understanding adverse effects of human-induced pollutants on descendant health and fitness—a research area that is also covered in ecology and evolution, biomedical science, and cross-disciplinary evidence syntheses (Fig. 7). Indeed, toxicology evidence syntheses covered primary literature on surprisingly diverse taxonomic groups (birds, fish, gastropods, and mammals) (Fig. 6B) which would suggest that literature on these taxonomic groups by other disciplines may be relevant (also see [32] for a systematic map that found a similar pattern for primary studies on non-genetic paternal effects from toxicology). Surprisingly, while agriculture tended to exhibit similar biases in research patterns to biomedical sciences (Fig. 7), agricultural evidence syntheses shared over 50% of their references with other disciplines (Fig. 9). This suggests that agricultural evidence syntheses cite a wider range of relevant literature compared to biomedical studies.

### Mapping the quality of studies relevant to the questions

#### Question four: critical appraisal

**Fig. 10:**
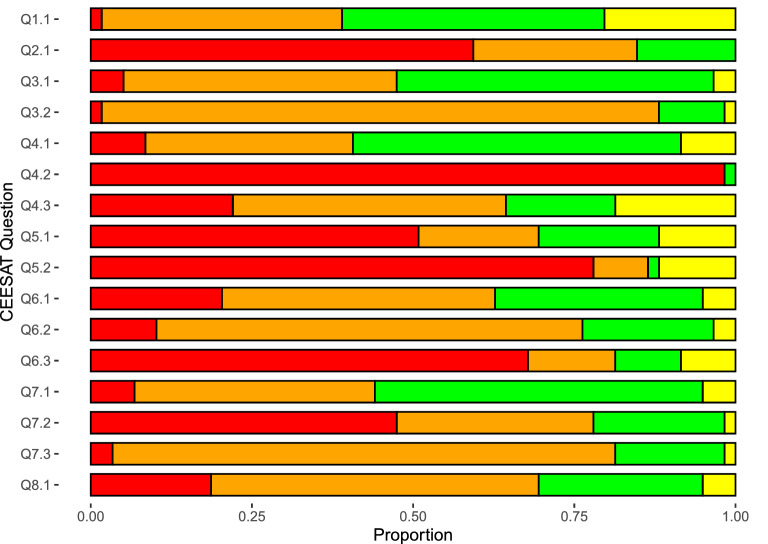
Average CEESAT scores across evidence syntheses. Yellow = gold score, green = green score, orange = amber score, and red = red score. Gold is the highest score, green is the second-highest score, amber is the second-lowest score, and red is the lowest score. See Additional file 3 for individual questions and requisites for each score

Our critical appraisal of SR quality using the Collaboration for Environmental Evidence Synthesis Assessment Tool (CEESAT) [34] showed some clear areas of the evidence syntheses that are mostly done to a high quality, and other areas that need improvement (Fig. 10; https://osf.io/8q3a9/ Additional file 5: Fig. S5, for individual study scores and Additional file 3 for a full description of CEESAT questions and scoring requirements). Specifically, the assessment questions that received the most green and gold scores asked if the elements of the SR were clear (question 1.1), if the eligibility criteria were clearly defined (question 4.1), and if the choice of synthesis approach was appropriate (question 7.1). This means that most evidence syntheses used a PICO/PECO/PO/PIT framework [78], it was clear how the evidence syntheses included or excluded primary research, and that meta-analysis/quantitative analysis was used when appropriate. In contrast, the assessment questions that largely received red scores asked if the eligibility criteria were consistently applied to all potentially relevant primary studies. Specifically, most evidence syntheses either did not report the number of reviewers performing the screening of the primary studies, or only one reviewer applied the eligibility criteria to potentially relevant studies (i.e., the inclusion or exclusion decisions were not cross-checked), consistency of decisions were not reported/tested when there was more than one reviewer, or the SR did not provide clear eligibility criteria. Furthermore, over 50% of evidence syntheses received an amber or red score for a majority of CEESAT questions (Fig. 10). This scoring is consistent with other reviews published in environmental science where methods are often not strongly systematic or the methods are not clearly reported [79, 80]. Such results are also consistent with a recent assessment of syntheses from the Collaboration for Environmental Evidence Database of Evidence Reviews (CEEDER) where it was found that many syntheses had issues with transparency and risk of bias [34]. These issues in reporting and methodology have implications for reproducibility and reliability of research and are also likely to impact for the benefit of policies that rely on robust research. Thus, there is also substantial room for improvement in the rigor and quality of the non-genetic inheritance SR literature.

### Limitations

Naturally there are limitations to our map due to the availability and reporting of the included articles as well as our own methodologies. We highlighted biases in research topics within and between disciplines. Specifically, there were taxonomic biases, as well as a strong bias towards inter-generational effects, particularly conferred through the matriline, effects of diet, and environmental effects during gestation. While these biases are likely to be partially linked to biases in the primary literature (similar patterns have been shown in [32, 81, 82]), and potentially a time-lag in research topics, there are some clear gaps in the evidence synthesis literature that should be filled. For example, the primary literature has shown that non-genetic effects through the patriline are common [47, 49–53], and that non-genetic effects can occur beyond the generation directly exposed to ancestral condition (trans-generational effects) [43–48], yet there are few evidence syntheses that synthesize such effects.

Additionally, our ability to extract and synthesize research patterns is based on the clear reporting within the relevant articles. For example, we often categorized articles as ‘unclear’ for many of our data elements if we were unable to determine specific details. This point also relates to the critical appraisal scoring where there were many methodological aspects of the SR methodology and reporting that require improvement. Furthermore, we show that there is a need for greater consistency in terminology use, and that it would be beneficial for the terms ‘inter-generational’ and ‘trans-generational’ to be used more frequently and consistently to make finding relevant literature easier [83].

Lastly, we must also acknowledge that we created research pattern and discipline categories in the most logical way based on our understanding of the literature, but results may differ slightly if different ways if splitting the categories were used. Furthermore, our ability to find relevant evidence syntheses may have been limited by our search strategy. For example, our literature search was conducted in English, and while we included other languages during literature screening (Japanese, Polish, Russian), the inclusion of only English search terms may have biased the literature towards studies published in English. In fact, the inclusion of only studies published in English can increase the risk of bias [75] and a substantial portion of the non-genetic evidence synthesis literature may be in languages other than English. The inclusion of this literature in our map would provide an even clearer understanding of the current state of the non-genetic inheritance evidence synthesis literature while also highlighting articles that may not always be easily located [74]. Furthermore, due to the many terms used to refer to non-genetic inheritance, we acknowledge that we may have missed some search terms (e.g., terminology that is specific to plants).

## Conclusions

### Implications for policy/management

We show that there is now a substantial body of work that systematically synthesizes the non-genetic inheritance literature, and this research has been increasing consistently over the last decade. This literature includes hundreds of species (many of which are key agricultural and fisheries species, those that are common for pre-clinical research, and those that signal ecosystem health). These species also come from diverse taxonomic groups, and the evidence syntheses span a range of environmental exposures, descendant traits, and ancestral and descendant sexes. Presenting these research patterns within and between disciplines allows policymakers to gain clear insights into the state of the literature and where to find relevant research. These insights can aid in making informed policy decisions relating to environmental pollutants, climate change, diseases and syndromes which has implications for population viability and agricultural production. It also points policymakers to research ‘hubs’ (e.g., the most productive authors shown in Additional file 5: Fig. S3) that policymakers can look to and contact for relevant research, and helps them to understand the overall quality and reliability of the current systematic-like literature. Highlighting the biases in the literature may also help to secure funding for understudied areas.

### Implications for research

Overall, we highlight within- and between-disciplinary research patterns that will enable researchers to work towards filling the gaps in the literature as well as pointing researchers in the direction of evidence syntheses (and primary literature) that may have otherwise been overlooked. In particular, we show that there is substantial scope to conduct evidence syntheses on non-genetic paternal effects with stable inheritance beyond the generation directly exposed to parental/ancestral condition (i.e., trans-generational inheritance), as well as to incorporate a wider range of environmental exposures and descendant traits. Additionally, using consistent terminology as well as creating greater connectedness among disciplines (e.g., citing literature beyond that specific to a discipline) would result in a greater cross-fertilization of ideas and make it easier for researchers to locate relevant literature beyond their specialization. Researchers should also aim to raise the quality of evidence syntheses in the areas that predominantly scored red and amber in the critical appraisal, as well as increase the connectedness of the literature by searching for relevant literature beyond their research field and aiming to form more diverse collaborations.

## Supplementary Information

**Additional file 1: **ROSES form.

**Additional file 2: Fig. S1.** Literature screen decision tree. Figure taken from our published protocol [1]. **Table S1. **Articles excluding during full-text screening and reason for exclusion.

**Additional file 3: **CEESAT questions and criteria.

**Additional file 4: **Data including included studies.

**Additional file 5:** Found at https://osf.io/8q3a9/**Fig. S2.** Filial generations within and between disciplines. Bar chart showing percent of SRs within and between generations that included primary studies of ancestral environmental effects on descendant filial generations. **Fig. S3.** Most productive authors over time. Figure showing the 15 most productive authors over time to publish SRs on non-genetic inheritance. The size of the blue cirles represents the number of articles publishes that year (“N. Articles”) and the transperancy of the blue circles represents the total citations per year (“TC per Year”). **Fig. S4.** Word cloud across disciplines. Word cloud of the most common key words across disciplines. **Fig. S5.** CEESAT individual scores. Individual CEESAT scores across SRs. Red = red score, orange = amber score, green = green score, yellow= gold score. See Appendix S2 for individual questions and requisites for each score.

## Data Availability

All data generated and code are available on the Open Science Framework at https://osf.io/8q3a9/ and on Github at https://github.com/elmacartney/Nongen_map_of_reviews
