# Supplementary material for: Evidence base for non-genetic inheritance of environmental exposures in non-human animals and plants: a map of evidence syntheses with bibliometric analysis
Source: Environ Evid. 2023 Jan 6;12:1. doi: 10.1186/s13750-022-00290-y (PMC11378868; doi:10.1186/s13750-022-00290-y)
Supplement: Supplementary file 2 — Additional file 2: Fig. S1. Literature screen decision tree. Figure taken from our published protocol [1]. Table S1. Articles excluding during full-text screening and reason for exclusion. [file 13750_2022_290_MOESM2_ESM.docx]

**Additional file 2**

**Appendix S2**

**Search strings:**

Web of Science Core Collection: TS=(( “silver spoon” OR “epigenetic inheritance” OR “non-genetic inheritance” OR “nongenetic inheritance” OR “extended inheritance” OR “extended heritability” OR “developmental programming” OR “developmental* program*” OR “DOHAD” OR *maternal* OR *paternal* OR *parental* OR trans-generation* OR multi-generation* OR inter-generation* OR across-generation* OR between-generation* OR transgeneration* OR multigeneration* OR intergeneration* OR epigenet* ) AND (( meta-analy* OR metaanaly* OR meta-regress* OR metaregress*) OR (systematic* OR comprehensiv* OR rapid OR scoping OR quantitativ* ) NEAR/5 ( review* OR map* OR synthes*)) NOT ( sport* OR econom* OR business* OR software* OR dent* OR orthodont* OR healthcare* OR patient* OR guideline* OR job* OR worker* OR veteran* OR school* OR student* OR child* OR infant* OR baby OR woman OR women OR breast* OR obstetr* OR eclampsia OR family OR placebo OR cancer OR violen* OR hospital* OR diagnos* OR autis* OR educat* OR countries OR china OR africa OR cohort* OR longit* OR rct OR qtl OR gwas OR genome-wide OR age* OR aging OR polyandr* OR chromosom* OR allel* OR genom* OR mutant OR polymorphism OR lifestyle OR leadership OR survey OR comment* OR corrigendum OR erratum ) )

Scopus: ( TITLE-ABS-KEY ( "silver spoon"  OR  "epigenetic inheritance"  OR  "non-genetic inheritance"  OR  "nongenetic inheritance"  OR  "extended inheritance"  OR  "extended heritability"  OR  "developmental programming"  OR  "developmental* program*"  OR  "DOHAD"  OR  *maternal*  OR  *paternal*  OR  *parental*  OR  trans-generation*  OR  multi-generation*  OR  inter-generation*  OR  across-generation*  OR  between-generation*  OR  transgeneration*  OR  multigeneration*  OR  intergeneration*  OR  epigenet* )  AND  ( TITLE-ABS-KEY ( meta-analy*  OR  metaanaly*  OR  meta-regress*  OR  metaregress* )  OR  TITLE-ABS-KEY ( ( systematic*  OR  comprehensiv*  OR  rapid  OR  scoping  OR  quantitativ*  OR  evidence )  W/5  ( review*  OR  map*  OR  synthes* ) ) )  AND NOT  TITLE-ABS-KEY ( sport*  OR  econom*  OR  business*  OR  software*  OR  dent*  OR  orthodont*  OR  healthcare*  OR  patient*  OR  guideline*  OR  job*  OR  worker*  OR  veteran*  OR  school*  OR  student*  OR  child*  OR  infant*  OR  baby  OR  woman  OR  women  OR  breast*  OR  obstetr*  OR  eclampsia  OR  family  OR  placebo  OR  cancer  OR  violen*  OR  hospital*  OR  diagnos*  OR  autis*  OR  educat*  OR  countries  OR  china  OR  africa  OR  cohort*  OR  longit*  OR  rct  OR  qtl  OR  gwas  OR  genome-wide  OR  age*  OR  aging  OR  polyandr*  OR  chromosom*  OR  allel*  OR  genom*  OR  mutant  OR  polymorphism  OR  lifestyle  OR  leadership  OR  survey  OR  comment*  OR  corrigendum  OR  erratum ) )

Pubmed: ("silver spoon"[Title/Abstract]) OR ("epigenetic inheritance"[Title/Abstract]) OR ("non-genetic inheritance"[Title/Abstract]) OR ("nongenetic inheritance"[Title/Abstract]) OR ("extended inheritance"[Title/Abstract]) OR ("extended heritability"[Title/Abstract]) OR ("developmental* program*"[Title/Abstract]) OR (DOHAD[Title/Abstract]) OR (*maternal*[Title/Abstract]) OR (*paternal*[Title/Abstract]) OR (*parental*[Title/Abstract] ) OR (trans-generation*[Title/Abstract]) OR (multi-generation*[Title/Abstract]) OR (inter-generation*[Title/Abstract]) OR (across-generation*[Title/Abstract]) OR (between-generation*[Title/Abstract] ) OR (transgeneration*[Title/Abstract]) OR (multigeneration*[Title/Abstract]) OR (intergeneration*[Title/Abstract]) OR (epigenet* [Title/Abstract]) Filters: Meta-Analysis, Systematic Review, Other Animals

BASE: Title: maternal OR paternal OR non-genetic OR nongenetic OR inter-gen OR intergen OR trans-gen OR transgen

Title: systematic OR meta-analysis OR metaanalysis

**
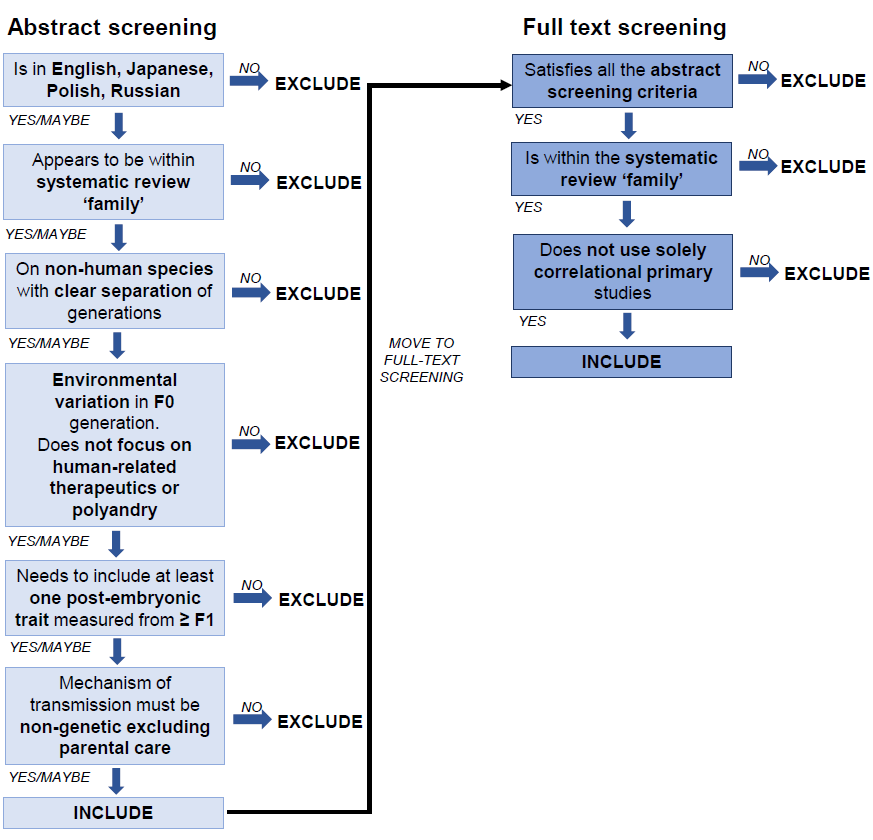
**

**Fig. S1:** Literature screen decision tree. Figure taken from our published protocol [1]

**Table S1** Articles excluding during full-text screening and reason for exclusion.

| **Article** | **Reason for exclusion** |
| --- | --- |
| Ajuogu, P. K., Wolden, M., McFarlane, J. R., Hart, R. A., Carlson, D. J., Van der Touw, T., & Smart, N. A. (2020). Effect of low-and high-protein maternal diets during gestation on reproductive outcomes in the rat: a systematic review and meta-analysis. Journal of animal science, 98(1). | No post-embryonic trait |
| Almeida, L. Z., Hovick, S. M., Ludsin, S. A., & Marschall, E. A. (2021). Which factors determine the long‐term effect of poor early‐life nutrition? A meta‐analytic review. Ecosphere, 12(8). | No F0 environmental variation |
| Badihian, N., Daniali, S. S., & Kelishadi, R. (2020). Transcriptional and epigenetic changes of brain derived neurotrophic factor following prenatal stress: A systematic review of animal studies. Neuroscience & Biobehavioral Reviews, 117, 211-231. | Conference abstract for a duplicate of included fulltext |
| Chastain, L. G., & Sarkar, D. K. (2017). Alcohol effects on the epigenome in the germline: Role in the inheritance of alcohol-related pathology. Alcohol, 60, 53-66. | Not within SR family |
| Derry, A. M., Fraser, D. J., Brady, S. P., Astorg, L., Lawrence, E. R., Martin, G. K., ... & Crispo, E. (2019). Conservation through the lens of (mal) adaptation: Concepts and meta‐analysis. Evolutionary Applications, 12(7), 1287-1304. | No experimental studies |
| Eirin-Lopez, J. M., & Putnam, H. M. (2018). Marine environmental epigenetics. Annual review of marine science, 11(1). | Not within SR family |
| Fitzgerald, E., Parent, C., Kee, M. Z., & Meaney, M. J. (2021). Maternal distress and offspring neurodevelopment: Challenges and opportunities for pre-clinical research models. Frontiers in Human Neuroscience, 15. | Not within SR family |
| Hammer, B., Wagner, C., Divac Rankov, A., Reuter, S., Bartel, S., Hylkema, M. N., ... & Krauss‐Etschmann, S. (2018). In utero exposure to cigarette smoke and effects across generations: a conference of animals on asthma. Clinical & Experimental Allergy, 48(11), 1378-1390. | Not within SR family |
| Harvey, N., Holroyd, C., Ntani, G., Javaid, K., Cooper, P., Moon, R., ... & Cooper, C. (2013). Maternal Pregnancy Vitamin D Status and Offspring Bone Health: A Systematic Review and Meta-analysis. Journal of Bone and Mineral Research, 28. | Fulltext unavailable |
| Hitchcock, D. J., Varpe, Ø., Andersen, T., & Borgå, K. (2017). Effects of reproductive strategies on pollutant concentrations in pinnipeds: A meta‐analysis. Oikos, 126(6), 772-781. | No experimental studies |
| Hur, S. S., Cropley, J. E., & Suter, C. M. (2017). Paternal epigenetic programming: evolving metabolic disease risk. Journal of Molecular Endocrinology, 58(3), 159-168. | Not within SR family |
| Jablonka, E., & Raz, G. (2009). Transgenerational epigenetic inheritance: prevalence, mechanisms, and implications for the study of heredity and evolution. The Quarterly Review of Biology, 84(2), 131-176. | Not within SR family |
| Marino, A. A. (1990). Meta-analysis of multi-generational studies of mice exposed to power-frequency electric fields. Journal of Bioelectricity, 9(2), 213-231. | Not within SR family |
| Marty, C., & BassiriRad, H. (2014). Seed germination and rising atmospheric CO 2 concentration: A meta‐analysis of parental and direct effects. New Phytologist, 202(2), 401-414. | Not within SR family |
| Mustieles, V., d'Cruz, S. C., Couderq, S., Rodríguez-Carrillo, A., Fini, J. B., Hofer, T., ... & David, A. (2020). Bisphenol A and its analogues: A comprehensive review to identify and prioritize effect biomarkers for human biomonitoring. Environment international, 144. | Not on animals |
| Neitzke, U. T. A., Harder, T.,I& Plagemann, A. (2011). Intrauterine growth restriction and developmental programming of the metabolic syndrome: a critical appraisal. Microcirculation, 18(4), 304-311. | Not within SR family |
| Ornellas, F., Carapeto, P. V., Mandarim-de-Lacerda, C. A., & Aguila, M. B. (2017). Obese fathers lead to an altered metabolism and obesity in their children in adulthood: review of experimental and human studies☆. Jornal de pediatria, 93, 551-559. | Not within SR family |
| Palma-Gudiel, H., Córdova-Palomera, A., Eixarch, E., Deuschle, M., & Fananas, L. (2015). Maternal psychosocial stress during pregnancy alters the epigenetic signature of the glucocorticoid receptor gene promoter in their offspring: a meta-analysis. Epigenetics, 10(10), 893-902. | Not on animals |
| Pei, Y., Forstmeier, W., & Kempenaers, B. (2020). Offspring performance is well buffered against stress experienced by ancestors. Evolution, 74(7), 1525-1539. | Not within SR family |
| Pettersen, A. K., White, C. R., Bryson‐Richardson, R. J., & Marshall, D. J. (2019). Linking life‐history theory and metabolic theory explains the offspring size‐temperature relationship. Ecology Letters, 22(3), 518-526. | No F0 environmental variation |
| Schwindt, A. R. (2015). Parental effects of endocrine disrupting compounds in aquatic wildlife: is there evidence of transgenerational inheritance? General and Comparative Endocrinology, 219, 152-164. | Not within SR family |
| Turecki, G., & Meaney, M. J. (2016). Effects of the social environment and stress on glucocorticoid receptor gene methylation: a systematic review. Biological Psychiatry, 79(2), 87-96. | No F0 environmental variation |
| Wang, Z., Zhang, T., Wu, J., Wei, X., Xu, A., Wang, S., & Wang, Z. (2021). Male reproductive toxicity of perfluorooctanoate (PFOA): Rodent studies. Chemosphere, 270. | No relevant descendant trait |
| Wong, M. G., The, N. L., & Glastras, S. (2018). Maternal obesity and offspring risk of chronic kidney disease. Nephrology, 23, 84-87. | Not within SR family |
| Xavier, M. J., Roman, S. D., Aitken, R. J., & Nixon, B. (2019). Transgenerational inheritance: how impacts to the epigenetic and genetic information of parents affect offspring health. Human Reproduction Update, 25(5), 519-541. | Not within SR family |
| Yan, X., Zhao, X., Li, J., He, L., & Xu, M. (2018). Effects of early-life malnutrition on neurodevelopment and neuropsychiatric disorders and the potential mechanisms. Progress in Neuro-Psychopharmacology and Biological Psychiatry, 83, 64-75. | Fulltext unavailable |
| Zhou, R., Lu, G., Yan, Z., Jiang, R., Bao, X., & Lu, P. (2020). A review of the influences of microplastics on toxicity and transgenerational effects of pharmaceutical and personal care products in aquatic environment. Science of the Total Environment, 732. | Not within SR family |
| Zhou, R., Lu, G., Yan, Z., Jiang, R., Bao, X., & Lu, P. (2020). A review of the influences of microplastics on toxicity and transgenerational effects of pharmaceutical and personal care products in aquatic environment. Science of the Total Environment, 732. | Not within SR family |

**Published protocol reference:**

1. Macartney EL, Drobniak SM, Nakagawa S, Lagisz M. Non-genetic inheritance of environmental exposures: a protocol for a map of systematic reviews with bibliometric analysis. Environmental Evidence. BioMed Central; 2021;10:1–11.
