# Supplementary material for: Evidence base for non-genetic inheritance of environmental exposures in non-human animals and plants: a map of evidence syntheses with bibliometric analysis
Source: Environ Evid. 2023 Jan 6;12:1. doi: 10.1186/s13750-022-00290-y (PMC11378868; doi:10.1186/s13750-022-00290-y)
Supplement: Supplementary file 3 — Additional file 3: CEESAT questions and criteria. [file 13750_2022_290_MOESM3_ESM.docx]

**Additional file 3**

**Appendix S3**

Collaboration for Environmental Evidence Synthesis Appraisal Tool (CEESAT) (Woodcock *et al.*, 2014). Version 2.1 updated in October 2020: https://environmentalevidence.org/wp-content/uploads/2021/02/CEESAT2-Version-2.1-updated-271020.pdf

|  | **The review question** |  |
| --- | --- | --- |
| 1.1 | Are the elements of the review clear? | - **Gold**: The review question or hypothesis is clearly stated and clearly defines key elements, (e.g., PICO, PECO, PO, PIT, etc.) correctly, such as the subject or population of interest, the intervention or exposure type, the comparator and valid measures of outcome.  - **Green**: The review question or hypothesis is clearly stated, and key-elements are mentioned although not formally defined in terms of PICO, PECO, PO, PIT, etc.  - **Amber**: The question or hypothesis is stated in broad terms but key-elements are unclear or poorly defined  OR  Question or hypothesis not stated but problem or issue is stated such that a question can be inferred.  - **Red**: A question, hypothesis or problem is not stated  OR  There is no stated objective to provide an answer to a question or test of a hypothesis  OR  The article does not contain an evidence synthesis (e.g., primary research or descriptive overview). |
|  | **The method/protocol** |  |
| 2.1 | Is there an a-priori method/protocol? | - **Gold**: The review cites a separate a-priori protocol or documented pre-defined method containing details of proposed conduct of all review and synthesis stages (e.g., question, search, eligibility screening, critical appraisal, data extraction and synthesis)  AND  It is linked from the synthesis (e.g., as supplementary material or hosted on a separate website)  AND  It was publicly accessible prior to the conduct of the review  AND  It was submitted to an independent body for peer review and publication.  **- Green**: The review cites a separate a-priori protocol or documented pre-defined  method containing details of conduct of all review and synthesis stages (e.g., question, search, screening, critical appraisal, data extraction and synthesis)  AND  It is linked from the synthesis (e.g., as supplementary material or hosted on a separate website)  AND  It was publicly accessible prior to the conduct of the review.  - **Amber**: The review cites a separate a-priori protocol or documented pre-defined method, but this does not contain all details of conduct of all review and synthesis  stages or was not publicly accessible prior to the conduct of the review  OR  The review includes a defined methods section (not a-priori) listing the synthesis stages conducted and providing sufficient detail to enable the method to be replicated (therefore this standard is met only if all of criteria 3.1, 4.1, 6.1 & 7.1 are rated green  or above).  - **Red**: There is no protocol and the review methods are not clearly defined in the methods section of the review or there are no methods reported. |
|  | **Searching for studies** |  |
| 3.1 | Is the approach to searching clearly defined, systematic and transparent? | - **Gold**: All search terms and search strings, with Boolean operators (‘AND’, ‘OR’ etc.) and wildcards, are clearly stated for each source (e.g., databases, search engines, specialist websites) so that the exact search is replicable by a third party  AND  There is information about the sources searched, together with dates of search and any limitations justified (e.g., languages, publication date, no grey literature searches).  - **Green**: All search terms and search strings, with Boolean operators (‘AND’, ‘OR’ etc.) and wildcards, are clearly stated for each major source (e.g., databases, search engines) so that the exact search is replicable by a third party but search terms for minor sources (e.g., specialist websites), if used, may be missing.  AND  There is information about the sources searched, together with dates of search but some limitations (reported or evident) not justified (e.g., languages or publication date or no grey literature searches)  - **Amber**: The search is described but not adequately to be fully replicable by a third party either because the specific search terms are not stated or Boolean  operators/wildcards are not stated (so it is unclear how the search terms are combined).  OR  There is information about the databases searched, but dates of search not given and no limitations justified (e.g., language or publication date or no grey literature  searches).  - **Red**: No information regarding the search strategy used. |
| 3.2 | Is the search comprehensive? | - **Gold**: Sources of articles searched capture both conventionally published scientific literature and grey literature using a combination of databases, search engines and specialist websites (may also be informed by stakeholders) or limitations are fully justified.  AND  Comprehensiveness of search is tested using independent samples of articles (test lists should be provided) of the relevant literature to demonstrate adequate sensitivity. NB. Statements such as ‘We considered only peer-reviewed material because this is more reliable than grey literature’ without evidence that the methodological quality of potentially relevant grey literature was assessed do not indicate that grey literature was objectively considered.  - **Green**: Sources of articles searched capture both conventionally published scientific literature and grey literature using a combination of databases, search engines and specialist websites (may also be informed by stakeholders)  OR  limitations are fully justified.  *NB. Statements such as ‘We considered only peer-reviewed material because this is more*  *reliable than grey literature’ without evidence that the methodological quality of potentially*  *relevant grey literature was assessed do not indicate that grey literature was objectively*  *considered.*  - **Amber**: Resources used are stated but limited, without justification, to conventionally  published scientific literature or just one or two sources.  - **Red**: Resources used not stated or search is not systematic (i.e., studies appear to have been selected). |
|  | **Including studies** |  |
| 4.1 | Are eligibility criteria clearly defined? | - **Gold**: Eligibility criteria are precisely defined (e.g., reliance on broad and potentially ambiguous terms should be avoided) and expressly related to each key element of the question (other criteria such as study design may also be considered)  AND  Criteria are consistent between a-priori protocol and review or differences are fully explained.  - **Green**: Eligibility criteria are precisely defined (e.g., reliance on broad and potentially ambiguous terms should be avoided) and are expressly related to each key element of the question (other criteria such as study design may also be considered).  **- Amber**: The questions/scope/objectives of the review are stated such that the type of primary research articles/studies to be included are broadly apparent, but the review does not explicitly identify criteria expressly related to each key element of the question  (other criteria such as study design may also be considered).  OR  Some eligibility criteria are defined but either incomplete or no clear relationship to a review question (possibly because the question is poorly defined).  - **Red**: No to both amber criteria above (eligibility criteria are not stated). |
| 4.2 | Are eligibility criteria consistently applied to all potentially relevant articles and studies  found during the search? | - **Gold:** The eligibility criteria are independently applied by more than one reviewer to all of the screened articles/studies (at title screening stage, pragmatic decisions about dual screening of subsamples is justified e.g., because large numbers of titles were  screened)  AND  Replicability of eligibility decisions was measured and reported and all disagreements between reviewers discussed so that the resolutions informed subsequent assessments.  **- Green**: The eligibility criteria are independently applied by more than one reviewer to a sample of justified size of the screened articles/studies at title, abstract and full text.  AND  Replicability of eligibility decisions was measured and reported and all disagreements between reviewers discussed so that the resolutions informed subsequent assessments.  **- Amber**: The eligibility criteria are applied by more than one reviewer to a sample of the screened articles/studies at abstract and full text but reviewer independence is uncertain (i.e., not reported) or absent.  AND  Replicability of eligibility decisions was measured and reported and all disagreements between reviewers discussed so that the resolutions informed subsequent assessments.  - **Red:** Number of reviewers not reported  OR  Only one reviewer applied criteria at abstract or full text stage,  OR  Where two reviewers, consistency of decisions not tested/reported  OR  No eligibility criteria provided (see 4.1) |
| 4.3 | Are eligibility decisions transparently reported? | - **Gold**: The number of unique articles found during the searches (after removal of duplicates) is presented  AND  The number excluded at each stage of the screening process is fully presented (e.g., in a flow diagram or table)  AND  Reasons for exclusion of each article/study considered at full text are presented (e.g. in an appendix)  AND  A list of eligible (included) articles/studies is presented (not just included in reference list).  - **Green**: The number of articles excluded at each stage of the screening process is reported but some aspects missing (e.g., number of unique articles or articles  unobtainable)  AND  Reasons for exclusion of each article/study considered at full text are presented (e.g. in an appendix)  AND  A list of eligible (included) articles/studies is presented (not just included in reference list).  - **Amber**: The number of articles excluded during the screening process is reported (or inferable) but some aspects missing (e.g., number of unique articles or articles unobtainable or reasons for exclusion at full text)  AND  A list of eligible (included) articles/studies is presented (not just included in reference list).  - **Red**: No to either or both of the amber criteria above |
|  | **Critical appraisal** |  |
| 5.1 | Does the review critically appraise each study? | - **Gold**: An effort is made to identify all sources of bias relevant to individual included studies (threats to internal and external validity)  AND  Each type of bias (threat to internal and external validity) is assessed and explained individually for all included studies  AND  Results are reported using an a-priori defined (in protocol) critical appraisal sheet.  *NB. This does not include syntheses in which the design and conduct of each study are stated*  *but validity is not explicitly considered (no critical appraisal), or in which methodological*  *rigour is discussed without transparent and objective assessments for each study.*  - **Green**: An effort is made to identify all sources of bias relevant to individual included studies (threats to internal and external validity)  AND  Each type of bias or threat to internal and external validity is assessed individually for all included studies and reported on a critical appraisal sheet.  *NB. This does not include syntheses in which the design and conduct of each study are stated but validity is not explicitly considered (no critical appraisal), or if which methodological rigour is discussed without transparent and objective assessments for each study.*  - **Amber**: Some characteristics of all included studies are explicitly identified as indicators of threats to internal and external validity of studies but not reported for individual studies.  - **Red**: No critical appraisal conducted  OR  all critical appraisal criteria not applied to all individual included studies. This may include syntheses in which the methods for each study are stated but validity is not  explicitly considered, or in which methodological rigour is discussed without transparent and objective assessments for each study. |
| 5.2 | During critical appraisal was an effort made to minimise subjectivity? | - **Gold**: An effort is made to minimise subjectivity by predefining a critical appraisal process in a protocol  AND  At least two people independently critically appraised each study with disagreements and process of resolution reported.  - **Green**: An effort is made to minimise subjectivity by predefining critical appraisal process in a protocol  AND  At least two people critically appraised each study but not independently (e.g., second person aware of first person’s decision)  - **Amber**: At least two people critically appraised each study but not independently (e.g. second person aware of first person’s decision) or subset of studies was appraised by at least two people independently. Disagreements and process of resolution MIGHT  NOT be reported.  - **Red**: No to Amber above (e.g., Only one person critically appraised each study or number not reported)  OR  No critical appraisal conducted (i.e., RED for 5.1 above). |
|  | **Data extraction** |  |
| 6.1 | Is the method of data extraction fully documented? | - **Gold**: The authors state in an a-priori protocol the type of data to be extracted  AND  the methods by which data from each study will be extracted so that the process can be replicated and confirm these methods were ultimately used in their report or reasons for deviation.  - **Green**: The authors state in the methods (but not in an a-priori protocol) the type of data to be extracted  AND  the methods by which data from each study were extracted so that the process can be replicated (In some cases methods may have been partially reported in an a-priori protocol but then modified or substantially developed during the review process).  - **Amber**: The authors state in the methods the type of data to be extracted  AND  although the review does not provide a fully replicable methodology for data extraction, it is possible to infer the broad method from the reported results (e.g., a  table that lists all eligible studies and data extracted might be included).  - **Red**: No to either part of amber above. It is not clear what data were selected for extraction and/or no consistent approach to data extraction is reported. |
| 6.2 | Are the extracted data reported for each study? | - **Gold**: All data selected for extraction are provided in a table or spreadsheet as set out in the a-priori protocol. This includes the data used in the synthesis from each primary study (e.g ‘raw’ outcome metrics: means, variance measures) and meta-data  AND  calculations or transformation of data by review authors using extracted data (e.g. effect sizes, averaging over variables summarisation of themes, coding, etc.) are  reported in full and therefore replicable.  - **Green**: All data selected for extraction provided in a post-hoc table or spreadsheet. This includes the data used in the synthesis from each primary study (e.g ‘raw’  outcome metrics: means and variance measures) and meta-data  AND  calculations or transformation of data by review authors using extracted data (e.g., effect sizes, averaging over variables summarisation of themes, coding, etc.) are  reported in full and therefore replicable.  - **Amber**: The review provides a table/spreadsheet that includes some of the extracted metrics data for some or all studies (e.g., Table/spreadsheet only lists partial data for each study but omits other information OR Table/spreadsheet lists extracted data, but  not for all studies OR A combination of these). Note: It may be unclear if all studies are included since they are not listed anywhere in the article.  - **Red**: No to amber above. Data extracted are not presented. |
| 6.3 | Were extracted data cross checked by more than one reviewer? | - **Gold**: Data were extracted from each study by at least two independent reviewers.  - **Green**: An explanation was provided of how a sample of extracted data was cross checked between two or more reviewers.  - **Amber**: A statement that cross-checking between two reviewers was carried out is provided but explanation unclear or incomplete.  - **Red**: No report of cross checking is provided. |
|  | **Data synthesis** |  |
| 7.1 | Is the choice of synthesis approach appropriate? | - **Gold**: The choice of synthesis method (i.e., quantitative or narrative synthesis) is prespecified,  described in sufficient detail to be replicable and justified (e.g., in the protocol) on the basis of scoping characteristics of included studies, taking into consideration variability between studies in sample size, study design, context, outcomes measures etc  OR  Justified post hoc as a deviation from the protocol (but still described in sufficient detail to be replicable) as a result of unexpected outcome (not pre-specified) of critical appraisal and data extraction  AND  Where quantitative and statistical approach to meta-analysis is not employed when it may have been appropriate, a justification for this should be given (e.g., studies too diverse or data not synthesisable).  - **Green**: The choice of synthesis method (i.e., quantitative or narrative synthesis) is described in sufficient detail to be replicable and is (or appears) justified on the basis of characteristics of included studies, taking into consideration variability between  studies in sample size, study design, context, outcomes etc.  AND  Where quantitative and statistical approach to meta-analysis is not employed when it may have been appropriate, a justification for this should be given (e.g., studies too diverse or data not synthesisable).  - **Amber**: No to either or both of the above (e.g., no justification for not undertaking metaanalysis when it may have been appropriate) but none of the listed criteria under Red below.  - **Red**: No to either or both in green above:  AND  Either quantitative synthesis (e.g., meta-analysis) is undertaken when inappropriate  OR  vote-counting relied on as an indicator of impact or effectiveness  OR  narrative synthesis does not include all studies or unclear if it includes all studies. |
| 7.2 | Is a statistical estimate of pooled effect (or similar) provided together with measure of  variance and heterogeneity among studies? | - **Gold**: Statistical estimates of findings are presented using pre-defined (e.g., in the protocol) meta-analysis method that justifies synthesis approach including study  weighting and subgroup analysis  AND  Consideration is given to study independence (e.g., through sensitivity analysis) and bias (e.g., tests for publication bias).  - **Green**: Statistical estimates of findings are presented using meta-analysis method that justifies approach (e.g., using study weighting and subgroup analysis)  AND  Consideration is given to study independence (e.g., through sensitivity analysis) and bias (e.g., tests for publication bias).  - **Amber**: Statistical estimates of findings presented using defined meta-analysis method but lacks justification of approach and/or consideration of study independence (e.g., through sensitivity analysis) and bias (e.g., tests for publication bias).  - **Red**: No statistical estimate provided either because meta-analysis not conducted or not possible  OR  Statistical estimate provided but not clear what meta-analysis method was used. |
| 7.3 | Is variability in the study findings investigated and discussed? | - **Gold**: A strategy for investigating effect modifiers is provided in an a-priori protocol and followed (or variations explained) in the review  AND  Effect modifiers (e.g., taxa being considered, location, habitat type, study design etc.) were investigated statistically through meta-analysis (alternatively, evidence for heterogeneity between studies is tested and reported as non significant)  AND  Authors have used results of critical appraisal (not just statistical weighting in metaanalysis) in their quantitative and/or narrative synthesis.  - **Green**: Effect modifiers (e.g., taxa being considered, location, habitat type, study design etc.) are investigated statistically through meta-analysis (alternatively,  evidence for heterogeneity between studies is tested and reported as non significant)  AND  Authors have used results of critical appraisal in their quantitative and/or narrative synthesis.  - **Amber**: Effect modifiers (e.g., taxa being considered, location, habitat type, study design etc.) investigated statistically through meta-analysis (alternatively, evidence for heterogeneity between studies is tested and reported as non-significant) but authors have not used results of critical appraisal in their quantitative and/or narrative synthesis.  OR  Effect modifiers (e.g., taxa being considered, location, habitat type, study design etc.) were investigated descriptively through narrative synthesis.  - **Red**: Reasons for variability in study findings not investigated. Effect modifiers were not considered (this includes studies that use quantitative synthesis but fail to test for heterogeneity statistically). |
|  | **Limitations** |  |
| 8.1 | Have the authors considered limitations in the synthesis? | - **Gold**: An explicit section is devoted to the authors’ consideration of limitations of their review including limitations of the primary data (available evidence), possible sources of bias in the review process, conduct of the review process and recommendations made for future syntheses and primary research.  - **Green**: An explicit section or identifiable passage of text is devoted to the authors’ consideration of limitations of primary research/data and of conduct of the review process but does not consider all of the following: possible sources of bias in the review process, conduct of the review process and recommendations made for future syntheses and primary research.  - **Amber**: Some consideration of limitations is evident but not explicitly stated or not focus of specific section  OR  Consideration of limitations of the primary research included but limitations of the conduct of the review not included.  - **Red**: No evident consideration of limitations of primary data or review conduct. |

**Reference**

Woodcock, P., Pullin, A.S. & Kaiser, M.J. 2014. Evaluating and improving the reliability of evidence syntheses in conservation and environmental science: a methodology. *Biol. Conserv.* **176**: 54–62. Elsevier.
